# Acute hiatal hernia: a late complication following gastrectomy

**DOI:** 10.1186/1755-7682-3-23

**Published:** 2010-10-04

**Authors:** Sara Piciucchi, Carlo Milandri, Giorgio Maria Verdecchia, Massimo Framarini, Elena Amadori, Manlio Monti, Devil Oboldi, Gianfranco Bandi, Domenico Barone, Giampaolo Gavelli

**Affiliations:** 1 Department of Radiology, IRST- Scientific Institute for study and treatment of Cancer, Meldola-Forlì; Italy; 2 Department of Oncology, IRST- Scientific Institute for study and treatment of Cancer, Meldola-Forlì; Italy; 3 Department of Advanced Oncological Surgery, Pierantoni-Morgagni Hospital, Forlì; Italy

## Abstract

**Introduction:**

We describe a case of acute hiatal hernia during chemotherapy, in a female patient previously treated with gastrectomy.

**Case presentation:**

After gastric resection, the patient underwent chemotherapy, developing important emetic symptoms. A radiograph of the abdomen was performed because of acute epigastrial pain and it showed a marked left diaphragm elevation.

A CT scan carried out 24 hours later identified an occlusion with herniation in the left hemi thorax. Subsequent surgical investigation resulted in a diagnosis of hiatal hernia with volvulus.

**Conclusions:**

This case represents a rare, late complication occurring after gastrectomy.

## Case presentation

A 47-year-old woman affected by tubular gastric adenocarcinoma (G3) with a poorly differentiated neuroendocrine component (N:7+/31) was treated with D2 gastrectomy in another hospital.

After surgical resection, total body computed tomography scan and tumor markers (CEA, CA 19-9 and NSE) were both negative.

Three months later, the patient was admitted in our institution to begin adjuvant chemotherapy with platinum, epirubicin, 5-fluorouracil and folic acid (PELF protocol) [1].

After the first two administrations, she was hospitalized because of increasing nausea and vomiting that did not respond to antiemetic drugs.

During hospitalization, the patient experienced epigastric pain that did not resolve with analgesic drugs and the vomiting persisted. Mild fever (38.5°C) was present, rapidly improving after the administration of amoxicillin and clavulanic acid.

**Figure 1:**
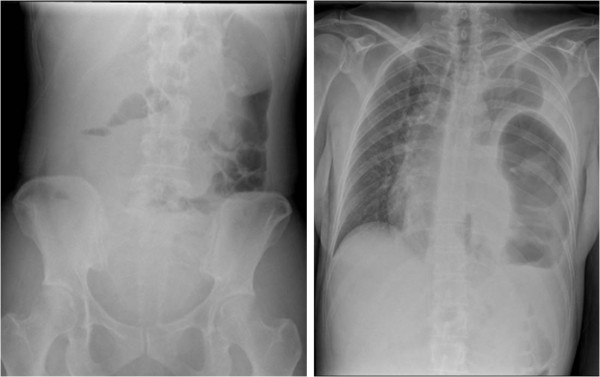
**Abdominal radiograph 24 hours later shows a marked increase of bowel distension in left hemi thorax, resulting in a compression of left parenchyma**. Descending and transverse colon is again distended. A mild fluid in the left pleural space is seen.

An abdominal X ray (Fig. 1) was performed to investigate the epigastrial pain.

The radiograph showed a moderate elevation of left hemi diaphragm associated to an intestinal air-fluid level.

Regular intestinal meteorism was absent, especially in the rectum and ascending colon.

Another radiograph was performed 24 hours later. It showed a significant increase of intestinal distension and the further elevation of the left hemi diaphragm with hypo expansion of left lung.

**Figure 2:**
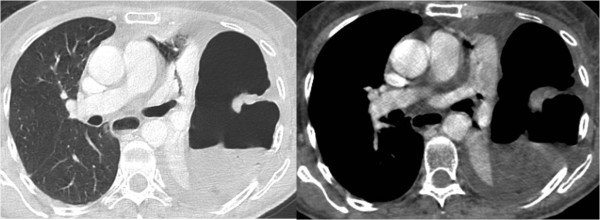
**CT scan at sub-carinal region shows air fluid level above diaphragm, indirect sign of a bowel herniation**. Moderate dilation of esophagus is visible (arrow) with fluid stagnation in the lumen. Partial collapse of left lower lobe and fluid in the pleural space is seen.

A moderate pleural effusion was observed in the left costophrenic angle. A urgent abdominal CT scan (Fig. 2) was performed that highlighted a giant air-fluid level occupying the left hemi thorax, with collapse of the lingula, the entire left lower lobe and part of the left upper lobe.

The remaining left upper lobe showed ground-glass opacity, induced by parenchymal congestion.

A moderate fluid was observed in the left pleural space. Marked fluid distension was seen in the ascending and transverse colon, with empty descending colon, sigma and rectum.

**Figure 3:**
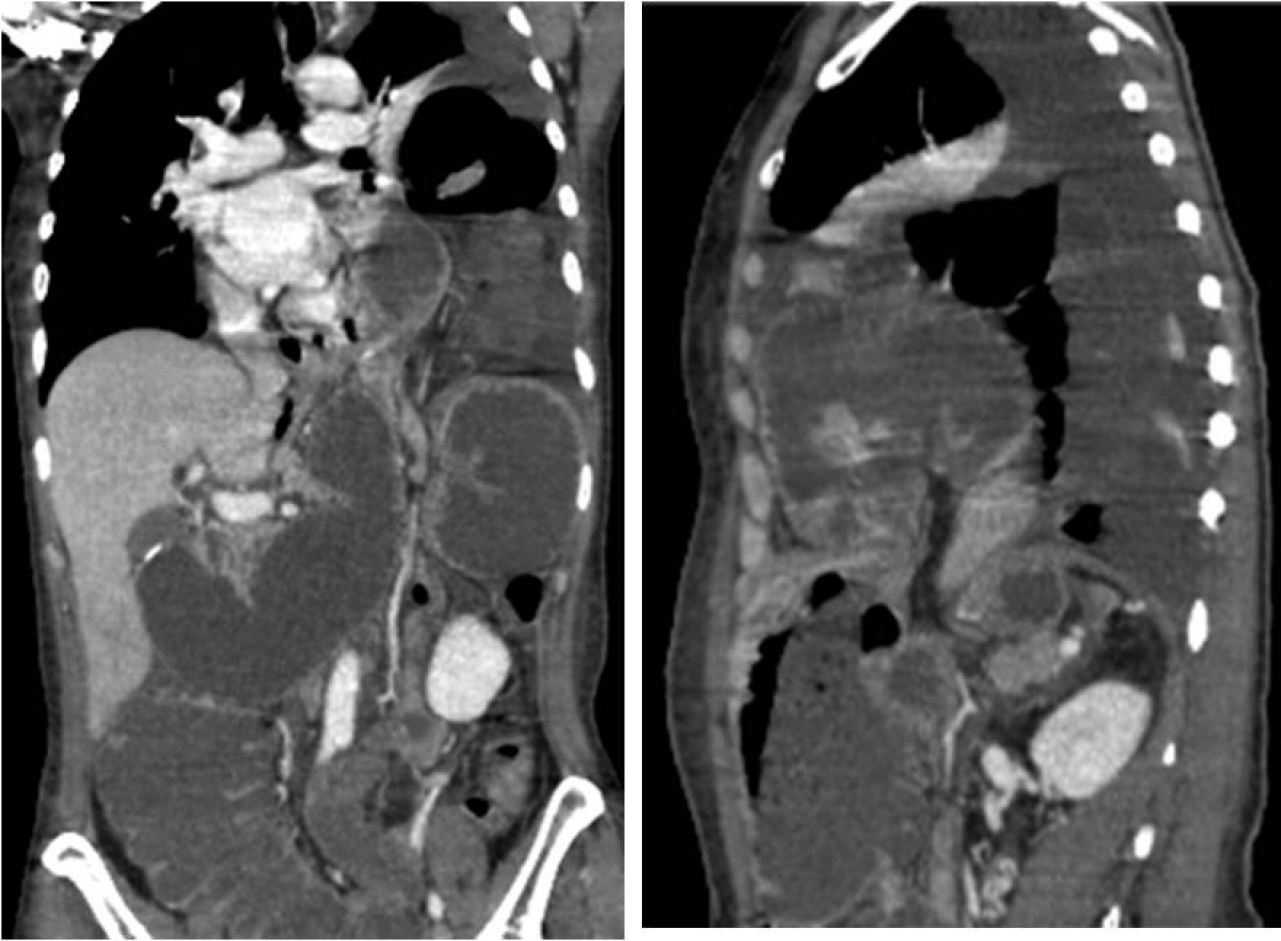
**Coronal and sagittal MPR reconstructions show abundant fluid distension of ascending and transverse colon**. In the sagittal view, tear of left diaphragm and herniation of intestinal loops are seen.

Coronal and sagittal multiplanar reformat (MPR) reconstructions (Fig. 3) confirmed abundant fluid distension of ascending and transverse colon.

In the sagittal view, a tear in the left diaphragm and herniation of intestinal loops were seen.

Radiological appearance was consistent with acute diaphragmatic hernia inducing a mechanical occlusion. The patient underwent urgent explorative laparotomy and final diagnosis was acute hiatal hernia with intestinal volvulus. It was probably related to a partial tear of the left diaphragmatic crus in the site of surgery, representing a late complication of partial gastrectomy. The acute herniation was likely consequence of an increased abdominal pressure, induced by vomiting.

Surgical repair of dome and crus of the left hemidiaphragm was performed. Post-surgical management showed left lung expansion, with a rapid decrease in pleural effusion.

The patient was discharged after seven days and three months later she was asymptomatic, with a normal chest X rays.

## Discussion

The diaphragm is a modified half-dome of musculofibrous tissue that separates the thorax from the abdomen. The thoracic side is covered with parietal pleura, and the abdominal side with peritoneum.

Four embryologic components arise during the formation of the diaphragm: the septum transversum, pleuroperitoneal folds, cervical myotomes, and the dorsal mesentery.

Injuries of diaphragm are recorded with a prevalence of 0.16-5% in blunt trauma patients.

Injuries are caused by a sudden increase in intra-abdominal or intrathoracic pressure against the fixed diaphragm.

The tears are typically large and involve the posterolateral surface of hemidiaphragm at the site of previous embryonic fusion. Injuries may occur at the central portion of the diaphragm or at the site of diaphragm attachments.

The surgical complications of conventional open surgery for gastric cancer, amply documented in the literature, are reported to be in the range of 17.4% and 46.0% [2-5].

Factors associated with surgical morbidity and mortality after open surgery include age, extent of lymph node dissection, combined resection, Billroth II reconstruction, duration of surgery and obesity. Major postoperative complications include anastomotic leak, pancreatic fistula, abdominal abscess and pneumonia.

Massive hiatal hernia with consequent volvulus represents a rare but serious condition that can result in intestinal strangulation. Generally, this clinical scenario is a late complication of a long-standing hiatal hernia.

In our patient, a partial surgical tear of the left diaphragmatic column induced an empty space into which the bowel herniated.

Diagnosis of diaphragmatic rupture depends on a high index of clinical suspicion and careful scrutiny of the chest X rays. In our clinical scenario we suspected an hernia because of the sudden elevation of left emidiaphragm with compression atelectasis in lower lobe and pleural effusion.

## Consent

Written informed consent was obtained from the patient for publication of this case report and accompanying images. A copy of the written consent is available for review by the Editor-in-Chief of this journal."

## Competing interests

The authors declare that they have no competing interests.

## Authors' contributions

SP; GG, AE, DB have analyzed the patient as regard radiological diagnosis; CM, MM: clinical management to case; GV, MF: surgical management. All authors read and approved the final manuscript
